# Our unique microbial identity

**DOI:** 10.1186/s13059-015-0664-7

**Published:** 2015-05-14

**Authors:** Jack A Gilbert

**Affiliations:** Argonne National Laboratory, University of Chicago, South Cass Avenue, Argonne, IL 60439 USA

## Abstract

A recent article examines the extent of individual variation in microbial identities and how this might determine disease susceptibility, therapeutic responses and recovery from clinical interventions.

Please see related article: http://dx.doi.org/10.1186/s13059-015-0646-9

## Background

What makes us human? Perhaps it is our individuality, our concept of self; in recent years, we have added to this concept by embracing the complexity of our microbial selves. Trillions of bacterial cells inhabit our body, outnumbering our own cells by an estimated factor of two or three. These bacterial cells also maintain a unique signature, which is most likely a result of each individual’s experiences and interactions with their environment. Just as no two people, even identical twins, share the exact same environmental experience, no two people are colonized by the same assemblage of microbial life. In a recent article for *Genome Biology*, Ana Zhu, Peer Bork and colleagues present compelling evidence to support the degree of this individual nature and discuss its clinical implications.

## Intra-species differences and bacteria-derived gene content in individual gut microbiomes

For the first time, Zhu *et al*. [1] utilized metagenomics data to quantify the degree of difference in gene content between strains of the same species of gut bacteria from different people. They examined 11 different bacterial species and, based on the degree of gene deletions, quantified the lower limit of gene variation at 13%. Interestingly, the most marked differences in gene functions among gut bacterial species related to polysaccharide utilization and polysaccharide capsid synthesis.

These findings suggest that to understand fully the role of the human microbiome in health and disease, we have to characterize it at the level of the taxonomic strain. A strain, in microbial ecology, refers to the taxonomic differentiation of organisms within a species. In bacteria, the level of genetic difference between strains of the same species can sometimes be dramatic, and can lead to significant changes in the phenotype of the microbe. Such changes could have profound influences on the phenotype of the host, possibly leading to changes in digestive ability, or in disease states, including those relating to autism, obesity and diabetes, among other conditions.

Recent work from Jill Banfield’s group at University of California, Berkeley has shown that inter-individual differences in the gut microbiome are apparent very soon after birth [2]. In this study, Raveh-Sadka and colleagues re-assembled hundreds of high-quality gut microbial genomes from infants in the same neonatal intensive care unit. Surprisingly, they found that there was very little sharing of bacteria between these infants. In fact, the strains of bacteria that colonized each child were unique. There are a number of reasons that might explain why this can occur.

First, it is possible that the children were colonized by bacteria specific to their mother: from her vaginal tract, from skin and oral contact, or through breast milk. A newborn infant is immediately colonized by microbes that are associated with the mode of delivery: either through the vagina or through the skin during cesarean section delivery [3]. Additionally, during the first year of life and sometimes beyond, a human child often receives milk from its mother, which we know maintains a complex microbiome originating from the mother’s gut and other mucosal surfaces [4]. Additionally, the mother’s own genome can shape the types of bacteria that can colonize breast milk [5], which comprises both a prebiotic of sugars, proteins and fats and a probiotic of bacterial organisms that transform foodstuffs into vital nutrients for the child. This would suggest that vertical transmission is an essential process for shaping our unique microbiome.

Second, the dispersal and interaction potential of microbes in the neonatal intensive care unit environment could be almost limitless, making the likelihood that any two infants would gain the same microbiome infinitesimally small. Additionally, host genetics can shape individual microbiomes [6]. Finally, the gut microbiome is a dynamic ecosystem, and as such is undergoing successional ecology. This means that when a new bacterium arrives, whether it colonizes or just passes through the ecosystem will depend on its phenotype and its interaction with any organisms already present there.

## The gut microbiome as an ecosystem

An ecosystem will constantly strive towards equilibrium or a stable state. Disturbances that alter this dynamic result in differences in the metabolic productivity and survival rate of different microbial species. Thinking in terms of the ecology of the human gut is one way to understand the complexity of the microbial milieu that occurs within our bodies [7]. The biogeography of the microorganisms that inhabit a person’s gut is similar to those of species that inhabit isolated islands, sometimes called ‘insular biogeography’ (also known as ‘island biogeography’). The eventual stable ecological state that defines either an island or a gut ecosystem is shaped by the dispersal, local diversification, environmental selection, and ecological drift of biological life interacting with a new environment.

A human child maintains a very unstable microbial ecosystem for approximately two or three years of its life [8]. This is an unusually long period of time based on what we know about microbial ecology. For example, a recent study in restroom surfaces demonstrated that the microbiome reached a stable state within 8 to 24 hours after initial colonization [9]. So why does the human microbiome take so long to reach stability? The most probable explanation is that the environment (the island in ecological terms) in which new microbes are arriving is constantly changing. The human body undergoes dramatic immunological, physiological and endocrinological changes during the first years of life; hence, to microbes living on and in it, the human body is like shifting sands. This dynamic feedback between the human body and its microbiome during these turbulent years probably leads to the selection of evolutionary traits that tend to stabilize the sources of microbes that an infant receives. The production of immunoglobulin A is key in this relationship because it has roles in keeping bacteria away from the cellular tissue in the gut while binding bacteria that are useful to the mucosal layer of the gut. This could be considered to be bacterial husbandry: management of the microbial milieu that interacts with the body. The human body, and probably the bodies of all other animal and possibly plants, has evolved mechanisms to recruit and retain microbes that have specific advantageous functions. These include microbes that can help to digest food, create certain products such as vitamin K, and protect against pathogens. The mechanisms for retaining advantageous microbes include vertical transmission from the parent to progeny, which provides the most likely explanation for early instances of individuality in the microbiome.

The individuality of microbial metabolism identified by Bork and colleagues using metagenomic data has specific implications for understanding how human lifestyle and habitat influence our microbial ecology. When metagenomic datasets from human microbiomes were compared with the genomes of isolated organisms, significantly higher variation in gene content was observed. Therefore, these metagenomic data captured a greater degree of metabolic diversity than can be found in the repertoires of genomes in the public repositories. This suggests that raw metagenomic data can help us to discover more diversity and more individuality in the human microbiome than can mapping metagenomic data to the known pan-genome diversity.

## Microbial diversity and human health

The use of metagenomics for the quantification of strain-level variance in our microbiota helps us to understand the unique differences in our lifestyle and ancestry that shape who we are. We are moving towards an era of personalized and precision medicine, in which we are becoming increasingly concerned about treating the whole individual so as to minimize the likelihood of adverse clinical outcomes, including non-response to therapeutic intervention. There is also the possibility for creating designer microbiomes that can be tailored to ensure a certain response or favorable outcome. Studies are needed to help create a public knowledge base that can be used to augment treatment strategies, develop novel probiotics, and assess the impact of lifestyle on our microbial ecology. In this light, crowd-funded and crowd-sourced efforts to catalogue the diversity of our microbiota, such as American Gut [10], are likely to become more commonplace in filling this knowledge gap. A better understanding of how the human microbiome varies between individuals will be essential for understanding differences in susceptibility to different diseases, differences in the response to a broad spectrum of therapeutics, and differences in how we recover from these interventions.

## References

[1] Zhu A, Sunagawa S, Mende D, Bork P (2015). Inter-individual differences in the gene content of human gut bacterial species. Genome Biol.

[2] Raveh-Sadka T, Thomas BC, Singh A, Firek B, Brooks B, Castelle CJ, et al. Gut bacteria are rarely shared by co-hospitalized premature infants, regardless of necrotizing enterocolitis development. eLife. 2015;4. doi:10.7554/eLife.05477. 10.7554/eLife.05477 PMC4384745 25735037

[3] Dominguez-Bello MG, Costello EK, Contreras M, Magris M, Hidalgo G, Fierer N (2010). Delivery mode shapes the acquisition and structure of the initial microbiota across multiple body habitats in newborns. Proc Natl Acad Sci U S A.

[4] Jeurink PV, van Bergenhenegouwen J, Jiménez E, Knippels LMJ, Fernández L, Garssen J (2013). Human milk: a source of more life than we imagine. Benef Microbes.

[5] Lewis ZT, Totten SM, Smilowitz JT, Popovic M, Parker E, Lemay DG, et al. Maternal fucosyltransferase 2 status affects the gut bifidobacterial communities of breastfed infants. Microbiome. 2015;3. doi:10.1186/s40168-015-0071-z. 10.1186/s40168-015-0071-z PMC4412032 25922665

[6] Goodrich JK, Waters JL, Poole AC, Sutter JL, Koren O, Blekhman R (2014). Human genetics shape the gut microbiome. Cell.

[7] Costello EK, Stagaman K, Dethlefsen L, Bohannan BJM, Relman DA (2012). The application of ecological theory toward an understanding of the human microbiome. Science.

[8] Koenig JE, Spor A, Scalfone N, Fricker AD, Stombaugh J, Knight R (2011). Succession of microbial consortia in the developing infant gut microbiome. Proc Natl Acad Sci U S A.

[9] Gibbons SM, Schwartz T, Fouquier J, Mitchell M, Sangwan N, Gilbert JA (2015). Ecological succession and viability of human-associated microbiota on restroom surfaces. Appl Environ Microbiol.

[10] American Gut. 2012. http://www.americangut.org. Accessed 27 April 2015.

